# Tricuspid Regurgitation Velocity and Mean Pressure Gradient for the Prediction of Pulmonary Hypertension According to the New Hemodynamic Definition

**DOI:** 10.3390/diagnostics13162619

**Published:** 2023-08-08

**Authors:** Giulia Elena Mandoli, Federico Landra, Benedetta Chiantini, Carlotta Sciaccaluga, Maria Concetta Pastore, Marta Focardi, Luna Cavigli, Flavio D’Ascenzi, Sonia Bernazzali, Massimo Maccherini, Serafina Valente, Matteo Cameli, Michael Henein

**Affiliations:** 1 Department of Medical Biotechnologies, Division of Cardiology, University of Siena, 53100 Siena, Italy; giulia.mandoli@unisi.it (G.E.M.); benedetta.chianti@student.unisi.it (B.C.); carlottasciaccaluga@gmail.com (C.S.); pastore2411@gmail.com (M.C.P.); focardim@unisi.it (M.F.); luna.cavigli@gmail.com (L.C.); flavio.dascenzi@unisi.it (F.D.); seravalente@gmail.com (S.V.); matteo.cameli@unisi.it (M.C.); 2 Institute of Public Health and Clinical Medicine, Umeå University, 90187 Umeå, Sweden; michael.henein@umu.se; 3 Department of Cardiac Surgery, University of Siena, 53100 Siena, Italy; s.bernazzali@gmail.com (S.B.); maccherini2@unisi.it (M.M.)

**Keywords:** pulmonary hypertension, echocardiography, right heart catheterization

## Abstract

Background: The hemodynamic definition of PH has recently been revised with unchanged threshold of peak tricuspid regurgitation velocity (TRV). The aim of this study was to evaluate the predictive accuracy of peak TRV for PH based on the new (>20 mmHg) and the old (>25 mmHg) cut-off value for mean pulmonary artery pressure (mPAP) and to compare it with the mean right ventricular–right atrial (RV–RA) pressure gradient. Methods: Patients with advanced heart failure were screened from 2016 to 2021. The exclusion criteria were absent right heart catheterization (RHC) results, chronic obstructive pulmonary disease, any septal defect, inadequate acoustic window or undetectable TR. The mean RV–RA gradient was calculated from the velocity–time integral of TR. Results: The study included 41 patients; 34 (82.9%) had mPAP > 20 mmHg and 24 (58.5%) had mPAP > 25 mmHg. The AUC for the prediction of PH with mPAP > 20 mmHg was 0.855 for peak TRV and mean RV–RA gradient was 0.811. AUC for the prediction of PH defined as mPAP > 25 mmHg for peak TRV was 0.860 and for mean RV–RA gradient was 0.830. A cutoff value of 2.4 m/s for peak TRV had 65% sensitivity and 100% positive predictive value for predicting PH according to the new definition. Conclusions: Peak TRV performed better than mean RV–RA pressure gradient in predicting PH irrespective of hemodynamic definitions. Peak TRV performed similarly with the two definitions of PH, but a lower cutoff value had higher sensitivity and equal positive predictive value for PH.

## 1. Introduction

Pulmonary hypertension is defined as an increase in mean pulmonary artery pressure (PAP) at rest. The diagnostic threshold of mean PAP has recently been lowered from 25 mmHg to 20 mmHg [1]. The decision of the ESC Task Force to lower the hemodynamic threshold for PH definition was driven by the need to increase the sensitivity of PH detection, to allow a timely initiation of specific therapies because of the prognostic impact of even a mildly increased mean PAP. This choice was supported by studies assessing the upper limit of normal PAP in healthy subjects [2,3,4] and by studies investigating the prognostic relevance of its increase [5,6,7]. Accurate diagnosis of pulmonary hypertension is of paramount importance in the context of advanced heart failure in order to determine the best management treatment, including heart transplantation [1].

Advanced heart failure patients are usually included in pulmonary hypertension clinical group 2, due to left heart disease [8]. Right heart catheterization is still the gold standard investigation for evaluating pulmonary circulation pressures and resistances [9]. Despite the unique value of right heart catheterization in ascertaining the final accurate diagnosis and identifying specific phenotypes of pulmonary hypertension, it is an invasive investigation with potential complications, such as vascular access-related complications, tricuspid valve injury, right ventricular perforation, infective endocarditis and arrhythmias [10]. Right heart catheterization is a valuable tool also for other clinical conditions, such as patients with adult congenital heart disease, cardiac evaluation for heart transplantation and left ventricular device implantation, and intracardiac shunt quantification. Thus, these complications limit its routine indications to only specific patients.

Current guidelines recommend the use of peak tricuspid regurgitation velocity (TRV), with a threshold of >2.8 m/s, instead of estimated systolic PAP in assessing the probability of the presence of pulmonary hypertension [11,12]. In fact, conflicting evidence has emerged regarding the accuracy of echocardiographic measures of PAP estimation in the last decade [13,14,15,16]. However, even though the hemodynamic definition of pulmonary hypertension has recently been revised, the threshold of peak TRV has remained unchanged at 2.8 m/s [11,15,17,18,19].

This study aimed to evaluate the predictive power of peak TRV for identifying patients with pulmonary hypertension classified according to the new (>20 mmHg) and the old (>25 mmHg) cutoff values of mean PAP and to compare the results with the mean right ventricular–right atrial (RV–RA) gradient, which ideally correlates better with mean PAP.

## 2. Materials and Methods

### 2.1. Patients Population

All consecutive patients with advanced heart failure undergoing right heart catheterization as part of the screening for heart transplantation eligibility from 2016 to 2021 at the University Hospital of Siena were retrospectively reviewed. The exclusion criteria were: right heart catheterization unavailability, chronic obstructive pulmonary disease, any septal defect, inadequate acoustic window or undetectable tricuspid regurgitation (TR). The study was conducted in accordance with the Declaration of Helsinki, and approved by the local Ethics Committee.

### 2.2. Data Collection and Echocardiography

Baseline characteristics, laboratory tests, echocardiographic and right heart catheterization data were retrospectively collected in a dedicated database.

All echocardiographic examinations were performed by experienced operators using GE Vivid E80/E95 echocardiographs equipped with an adult 1.5–4.3 MHz phased array transducer, with an ECG continuously monitored, according to the American Society of Echocardiography/European Association of Cardiovascular Imaging recommendations [20,21]. Complete echocardiographic examinations were performed just before right heart catheterization on the same day. TR was evaluated with color Doppler interrogation in apical four-chamber view. A clearly delineated continuous-wave (CW) Doppler TR signal was acquired for accurate calculation of peak TRV and velocity–time integral (VTI). Also, inferior vena cava cine registration during a complete respiratory cycle was a requirement to allow estimation of right atrial (RA) pressure using its diameter and collapsibility.

The mean right ventricular–right atrial (RV–RA) pressure gradient was retrospectively obtained from VTI of TR. The mean PAP was obtained by adding the mean RV–RA pressure gradient to estimated mean RA pressure [22]. The maximum RV–RA gradient was also retrospectively estimated from peak TRV by applying the modified Bernoulli equation (4V^2^). Systolic PAP was calculated by adding the maximum RV–RA pressure gradient to mean RA pressure. Diastolic PAP was derived from mean PAP and systolic PAP using the formula: diastolic PAP = 1.5 × [mean PAP—(systolic PAP/3)] [23]. All other standard and advanced (i.e., global longitudinal strain (GLS)) echocardiographic parameters were obtained according to the American Society of Echocardiography/European Association of Cardiovascular Imaging recommendations [20,21]. Particularly, linear internal measurements of the left ventricle were acquired from the parasternal long-axis view with 2D-guided images. Right ventricular mid-end-diastolic diameter was also obtained at the middle third of RV inflow, approximately halfway between the maximal basal diameter and the apex at end-diastole. The LV ejection fraction (LVEF) was calculated using the biplane method of disks summation (modified Simpson’s rule), while the RV fractional area change (FAC) was calculated using the formula: RV FAC (%) = 100 × (EDA − ESA)/EDA, where EDA is the end-diastolic area and ESA is the end-systolic area.

For speckle-tracking echocardiography analysis, endocardial borders and myocardium of all segments from the apical views (four chambers, two chambers and apical long axis) had to be clearly visualized throughout the whole cardiac circle. Analysis was retrospectively performed offline using EchoPAC software v204 (GE Healthcare) and conventional protocol [20]. 

### 2.3. Right Heart Catheterization

Right heart catheterization was performed on the same day as the echocardiographic examination, just after it. A pulmonary artery Swan–Ganz catheter was used to obtain mean RA pressure, diastolic, systolic and mean PAP and pulmonary arterial wedge pressure (PAWP), using conventional technique [24]. Pressure curves were recorded and selected by an experienced operator with the values averaged over ten heart beats, independent of the respiratory cycle. The cardiac index was derived by indexing cardiac output using the indirect Fick method for body surface area. Pulmonary resistances were indirectly calculated from pressure and flow data.

### 2.4. Statistical Analysis

Continuous data are presented as mean and standard deviation or as median and interquartile range, as appropriate. The Kolmogorov–Smirnov test was used to verify normal distribution of variables. Categorical data are summarized as absolute and relative frequencies. To assess the predictive performance of the echocardiographic variables for PH, Receiver Operating Characteristic (ROC) curves were created, and the Youden index was used to determine the best cutoff values. To evaluate the strength of the relationship between echocardiographic and RHC data, a correlation analysis using Pearson’s correlation coefficient was performed. To evaluate agreement between the two methods, a Bland–Altman analysis was carried out. A *p*-value < 0.05 was considered statistically significant. Analysis was performed using SPSS, version 26 (SPSS, Chicago, IL, USA).

## 3. Results

### 3.1. Patients Population

In the first instance, 91 patients were recruited in the study. Thirty-seven patients were excluded from the analysis because right heart catheterization was not performed at the time of data collection, 1 patient because of an interatrial septal defect, 3 patients because of chronic obstructive pulmonary disease and 9 patients because of inadequate acoustic window or undetectable TR. The final population included in the analysis was comprised of 41 patients.

The median age of patients was 58 years (IQR: 52–62), who were mostly men (82.9%). The etiology of heart failure was predominantly nonischemic (61.0%). Most patients had ICDs (85.4%). The median NT-proBNP was 1522 pg/mL (IQR: 649–2550). Most patients were in NYHA class II (25 patients, 61.0%) or III (10 patients, 24.4%). The complete patient’s baseline characteristics are presented in Table 1.

### 3.2. Echocardiographic Parameters

The mean left ventricular end-diastolic diameter was 68 ± 11 mm, and the median left ventricular ejection fraction (LVEF) was 28% (IQR: 23–35). The median right ventricular mid-end-diastolic diameter was 31 mm (IQR: 29–34), and the median fractional area change (RVFAC) was 38% (IQR: 33–45). Most patients had mild TR (51.3%), while the minority had severe TR (19.5%). Twelve patients had moderate TR (29.2%). The median peak TRV was 2.5 m/s (IQR: 2.2–3.0), and the median RV–RA gradient was 18 mmHg (10–24). The complete echocardiographic measurements are shown in Table 2.

### 3.3. Right Heart Catheterization

The mean RA pressure was 7.5 ± 4.1 mmHg. The mean systolic PAP was 39 ± 12 mmHg. The mean PAP was 27 ± 9 mmHg. The median PAWP was 17 mmHg (IQR: 10–22). The median pulmonary vascular resistance was 2.51 WU (IQR: 1.95–2.98). Thirty-four patients (82.9%) had mean PAP > 20 mmHg, and 24 patients (58.5%) had mean PAP > 25 mmHg. The complete right heart catheterization measurements are reported in Table 3.

### 3.4. Predictive Performance of Echocardiographic Measurements

**Figure 1:**
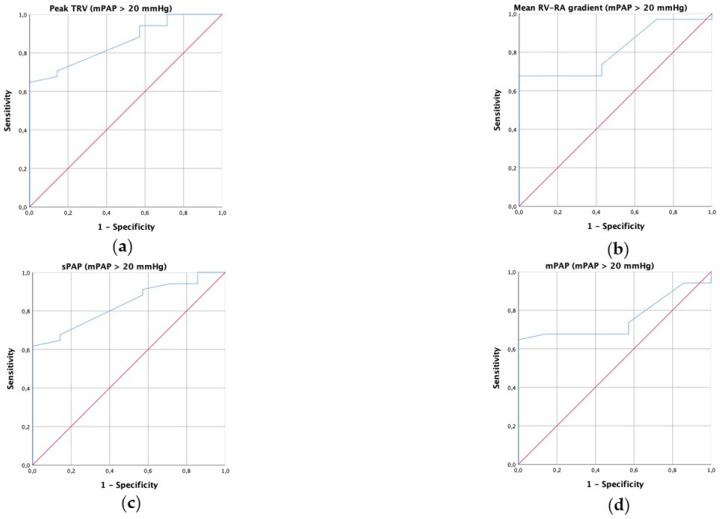
Receiver operating characteristic (ROC) curve for prediction of pulmonary hypertension defined as mean pulmonary artery pressure > 20 mmHg through peak tricuspid regurgitation velocity (TRV, (**a**)), mean right ventricular–right atrial (RV–RA) pressure gradient (**b**), estimated systolic pulmonary arterial pressure (sPAP, (**c**)) and estimated mean pulmonary arterial pressure (mPAP, (**d**)).

**Figure 2:**
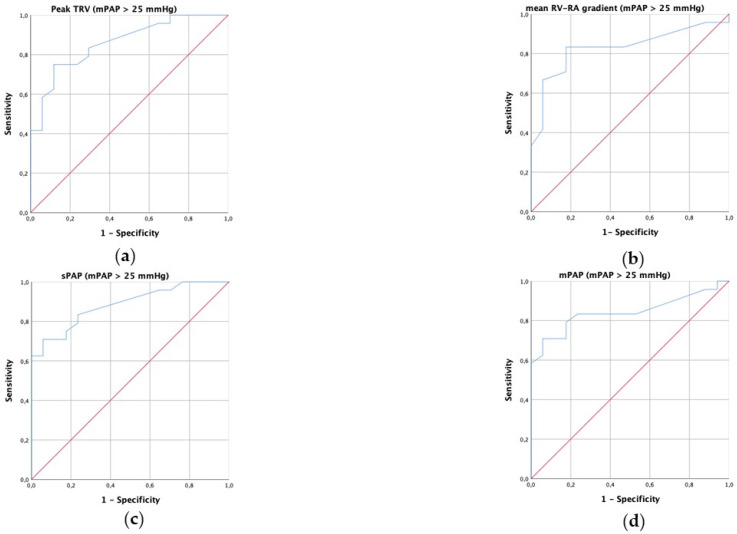
Receiver operating characteristic (ROC) curve for prediction of pulmonary hypertension defined as mean pulmonary artery pressure > 25 mmHg through peak tricuspid regurgitation velocity (TRV, (**a**)), mean right ventricular–right atrial (RV–RA) pressure gradient (**b**), estimated systolic pulmonary arterial pressure (sPAP, (**c**)) and estimated mean pulmonary arterial pressure (mPAP, (**d**)).

According to the new definition of PH (mPAP > 20 mmHg), the area under the ROC curve (AUC) for peak TRV in predicting PH was 0.855, for mean RV–RA gradient was 0.811, for estimated sPAP was 0.834 and for estimated mPAP was 0.758 (Figure 1). According to the old definition of PH (mPAP > 25 mmHg), the AUC for peak TRV in predicting PH was 0.860, for mean RV–RA gradient was 0.830, for estimated sPAP was 0.884 and for estimated mPAP was 0.843 (Figure 2). A peak TRV cutoff value of 2.4 m/s had 65% sensitivity and 100% positive predictive value for predicting PH (according to the new definition). The current cutoff of 2.8 m/s of peak TRV had 44% and 100% positive predictive value for predicting PH (according to the new definition).

### 3.5. Correlation between Echocardiographic Measurements and RHC

The correlation between peak TRV and RHC-derived sPAP was r = 0.624, *p* < 0.001. The correlation between mean RV–RA gradient and RHC-derived mPAP was r = 0.573, *p* < 0.001. The correlation between echocardiography-estimated PA pressures and RHC-derived PA pressures was: mPAP, r = 0.608, *p* < 0.001; sPAP, r = 0.616, *p* < 0.001 and dPAP, r = 0.576, *p* < 0.001.

**Figure 3:**
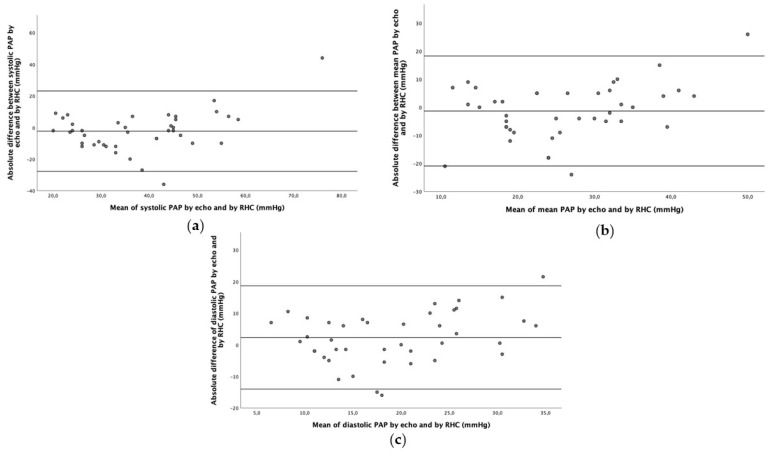
Absolute mean differences between pulmonary artery pressure by echocardiography (systolic, (**a**); mean, (**b**); diastolic, (**c**)) and right heart catheterization. PA, pulmonary artery; RHC, right heart catheterization.

The absolute mean difference between mean, systolic and diastolic PA pressures by RHC vs. echocardiography was −1.3 ± 9.8 mmHg, 2.4 ± 12.7 mmHg and −2.3 ± 8.2 mmHg, respectively. Figure 3 shows relative Bland–Altman plots.

## 4. Discussion

The main findings of this study can be summarized as follows: (1) Peak TRV performed similarly in predicting pulmonary hypertension according to the two hemodynamic definitions (old = mean PAP > 25 mmHg; new = mean PAP > 20 mmHg), but a lower cutoff value of 2.4 m/s had higher sensitivity and equal positive predictive value when applying the new definition of pulmonary hypertension, compared with the currently used threshold; (2) Peak TRV performed better than the mean RV–RA pressure gradient in predicting pulmonary hypertension for both old and new definitions; and (3) Estimation of pulmonary artery pressures using echocardiographic methods mainly based on TR analysis is modestly accurate when compared to right heart catheterization.

In the context of the evaluating pulmonary circulation hemodynamics, Doppler echocardiography still remains the first-line noninvasive investigation, being patient-friendly and an easy bed-side test [11]. However, the echocardiographic assessment carries various limitations, and awareness of potential pitfalls is important in order to avoid drawing the wrong diagnostic conclusions. Firstly, it must be remembered that echocardiography is able to provide only estimates of hemodynamic parameters. Additional signs of pulmonary hypertension are necessary to confirm the suspicion of the disease. Secondly, for PAP estimation, RA pressure needs also to be estimated [25]. Importantly, both a lesser degree of TR and severe TR may lead to underestimation of systolic PAP by peak TRV [23]. Similarly, RV systolic dysfunction may lead to inaccurate estimates. Thirdly, for mean PAP estimate from TR VTI, a complete TR envelope is necessary to avoid under- or overestimation [26]. Even though the hemodynamic diagnosis of pulmonary hypertension is made considering the mean PAP, current guidelines recommend the use of peak TRV instead of estimated systolic PAP when suspecting pulmonary hypertension [11]. Moreover, even though the hemodynamic definition of pulmonary hypertension has recently been revised, the threshold for peak TRV has remained unchanged [15,17,18,19].

Peak TRV is calculated from TR evaluated with CW Doppler interrogation of the tricuspid valve in apical four-chamber view. Applying Bernoulli’s equation, maximum and mean RV–RA gradients can be calculated from the same acquisition, considering peak TRV and from VTI calculation, respectively. Therefore, not only systolic PAP may be estimated from TR and estimated RA pressure, but also mean PAP and, consequently, diastolic PAP. Therefore, the aim of this study was to, firstly, evaluate if peak TRV performed well even according to the new hemodynamic definition of pulmonary hypertension, and, secondly, to evaluate if other TR-derived parameters could potentially perform better than peak TRV in predicting pulmonary hypertension.

Regarding the first question, in this study, we have shown that peak TRV performs well independently of the pulmonary hypertension definition used in an advanced heart failure population, with similar predictive performance according to the two definitions. However, in our study population, a lower cutoff value for peak TRV had a higher sensitivity but equal positive predictive value for pulmonary hypertension when the new hemodynamic definition is used as compared to the currently recommended threshold. As a first-line tool to assess the probability of pulmonary hypertension, sensitivity should be preferred over specificity to allow a higher detection of patients affected by pulmonary hypertension. Indeed, for the same reason, recent guidelines have lowered the cutoff of mean PAP from 25 to 20 mmHg to make a diagnosis of pulmonary hypertension. The information coming from additional echocardiographic parameters and second-level examinations with higher specificity will allow for recognizing false-positive patients resulting from the first assessment.

As already mentioned, peak TRV may be used to derive maximum RV–RA pressure gradient when using the modified Bernoulli’s equation, which ideally better reflects systolic PAP rather than mean PAP. In fact, mean PAP may be better reflected by mean RV–RA pressure gradient. Therefore, we evaluated the predictive performance of mean RV–RA pressure gradient for pulmonary hypertension, which, however, proved to be less accurate than peak TRV in predicting the presence of pulmonary hypertension, irrespective of the used definition, new or old. Also, estimated pulmonary artery pressures through adding estimated RA pressure to maximum and mean RV–RA pressure gradients, namely, systolic PAP and mean PAP, were generally inferior to peak TRV in predicting pulmonary hypertension. Curiously, in our study population, systolic PAP performed better then peak TRV in the prediction of pulmonary hypertension according to the old definition, but these data were not confirmed when using the new hemodynamic definition.

Finally, correlation analysis and Bland–Altman analysis showed only modest agreement between echocardiographically estimated PAPs and right heart catheterization-derived PAPs. The largest absolute mean difference and the highest dispersion were observed for systolic PAP. Therefore, the consistency of results regarding peak TRV further support its use results to assess the probability of having pulmonary hypertension instead of mean RV–RA pressure gradient and estimated systolic PAP and mean PAP, as recommended by current guidelines. However, our results pose an issue on the threshold of peak TRV when the new hemodynamic definition is used, at least in an advanced heart failure population. In fact, lowering the cutoff of peak TRV in light of the new hemodynamic definition of pulmonary hypertension should be taken into consideration due to the higher sensitivity and equal positive predictive value as compared to the current threshold. The results of this study provide new findings regarding the accuracy of commonly used echocardiographic tools according to both the new and the old hemodynamic definition of PH in patients with AHF, who are evaluated for heart transplantation. This is particularly valuable since PH is a key element to assess during the heart transplantation evaluation protocol. The results may appear to be in contrast to previous studies with similar purposes, even though a direct comparison cannot be made because of the significantly different populations [19].

### Limitations

This study is a single-center and data analysis was retrospective in nature, which are known for their limitations. The included patients are part of a very selected and restricted population of subjects with advanced heart failure, which may limit the extent of applying the results to the general population. However, since analyses were performed between measurements with the two methods in the same individual, the above concern is only modest. Even though the included patients belong to those with AHF, considered for heart transplantation, one of the main limitations of the study remains the relatively small sample size, which may limit the broad implication of the results. Also, because of the retrospective design of the study, we did not perform a sample size calculation and performed the analysis with the available data at our institution, which thereafter proved sufficient for the purpose of the study. We selected a population of patients with advanced heart failure because of the high prevalence of pulmonary hypertension in this group and the routine performance of right heart catheterization to assess eligibility for heart transplantation. Estimation of pulmonary pressures from TR can be affected by both TR degree and right ventricular impairment, both conditions are frequently seen in this study population of AHF patients. Despite this being a limitation, it allows for exploring the validity of the commonly used echocardiographic tools in screening for PH even in this special population of patients. Being a retrospective study, there were not enough echocardiographic exams with a CW Doppler registration at the pulmonary valve level, in order to estimate pulmonary artery pressures using pulmonary regurgitation signals. Moreover, the lack of pulsed-wave Doppler acquisitions of right heart including RV filling time and pulmonary acceleration time should be mentioned.

## 5. Conclusions

Peak TRV performed better than other echocardiographic variables for the prediction of pulmonary hypertension according to the new hemodynamic definition. Also, peak TRV performed similarly with the old definition of pulmonary hypertension, but a lower cutoff showed higher sensitivity and equal positive predictive value for pulmonary hypertension as compared to the current one.

## Figures and Tables

**Table 1:** Characteristics of the study population.

Variables	
Age (years)	58 (52–62)
Male gender (*n*, %)	34 (82.9%)
BSA (m^2^)	1.97 ± 0.21
Smokers (*n*, %)	17 (41.4%)
Dyslipidemia (*n*, %)	27 (65.9%)
Diabetes (*n*, %)	10 (24.4%)
Hypertension (*n*, %)	14 (34.1)
Obesity (BMI > 30 kg/mq)	11 (26.8%)
CKD (eGFR < 60 mL/min/1.73 mq; *n*, %)	8 (19.5%)
Atrial fibrillation (*n*, %)	16 (39.0%)
ICD (*n*, %)	35 (85.4%)
Etiology of HF	
Ischemic (*n*, %)	16 (39.0%)
Nonischemic (*n*, %)	25 (61.0%)
NYHA class	
Class I (*n*, %)	6 (14.6%)
Class II (*n*, %)	25 (61.0%)
Class III (*n*, %)	10 (24.4%)
Class IV (*n*, %)	0 (0.0%)
Hb (g/dL)	14.2 ± 1.5
WBC (×10^9^/L)	7.48 (6.38–9.24)
Creatinine (mg/dL)	1.14 ± 0.23
Bilirubin (mg/dL)	0.7 (0.4–0.9)
NT-proBNP (pg/mL)	1522 (649–2550)

Data are expressed as mean ± DS or median (IQR)]. BSA, body surface area; BMI, body mass index; CKD, chronic kidney disease; eGFR, estimated glomerular filtration ratio; COPD, chronic obstructive pulmonary disease; ICD, implantable cardioverter defibrillator; HF, heart failure; Hb, hemoglobin; WBC, white blood cells; NT-proBNP, N-terminal pro–B-type natriuretic peptide.

**Table 2:** Echocardiographic parameters.

Variables	
IVS (mm)	10 ± 2
EDD (mm)	68 ± 11
EDV (mL)	220 (169–309)
LVEF (%)	28 (23–35)
LA volume (mL)	126 (98–146)
Mid RV EDD (mm)	31 (29–34)
TAPSE (mm)	18 (14–20)
RV s’ (m/s)	0.10 (0.08–0.11)
RV FAC (%)	38 (33–45)
E/A	1.22 (0.70–2.34)
E/e’	13 (10–17)
MR	
Mild (*n*, %)	4 (9.8%)
Moderate (*n*, %)	8 (19.5%)
Severe (*n*, %)	11 (26.8%)
TR	
Mild (*n*, %)	21 (51.3%)
Moderate (*n*, %)	12 (29.2%)
Severe (*n*, %)	8 (19.5%)
AR	
Mild (*n*, %)	7 (17.1%)
Moderate (*n*, %)	3 (7.3%)
Severe (*n*, %)	1 (2.4 %)
PR	
Mild (*n*, %)	23 (56.1%)
Moderate (*n*, %)	5 (12.2%)
Severe (*n*, %)	0 (0.0%)
IVC (mm)	19 ± 4
RA pressure (mmHg)	5 (5–10)
Peak TRV (m/s)	2.5 (2.2–3.0)
sPAP (mmHg)	34 (25–45)
AcT (msec)	101 ± 27
Mean RV–RA gradient (mmHg)	18 (10–24)
dPAP (mmHg)	18 (10–29)
mPAP (mmHg)	23 (15–35)
LV GLS (%)	−6 (−8–−3)

Data are expressed as mean ± DS or median (IQR). IVS, interventricular septum; EDD, end-diastolic diameter; EDV, end-diastolic volume; LV, left ventricle; EF, ejection fraction; LA, left atrium; RV, right ventricle; TAPSE, tricuspid annular plane systolic excursion; FAC, fractional area change; MR, mitral regurgitation; TR, tricuspid regurgitation; AR, aortic regurgitation; PR, pulmonary regurgitation; IVC, inferior vena cava; RA, right atrium; sPAP, systolic pulmonary artery pressure; AcT, acceleration time; dPAP, diastolic pulmonary artery pressure; mPAP, mean pulmonary artery pressure; GLS, global longitudinal strain.

**Table 3:** Right heart catheterization parameters.

Variables	
Mean RA pressure (mmHg)	7.5 ± 4.1
Systolic PA pressure (mmHg)	39 ± 12
Diastolic PA pressure (mmHg)	19 (12–24)
Mean PA pressure (mmHg)	27 ± 9
Pulmonary arterial wedge pressure (mmHg)	17 (10–22)
Cardiac index (L/min/mq)	2.07 ± 0.38
Pulmonary vascular resistance (WU)	2.51 (1.95–2.98)

Data are expressed as mean ± DS or median (IQR). RA, right atrium; RV, right ventricle; PA, pulmonary artery.

## Data Availability

The data underlying this article will be shared on reasonable request to the corresponding author.
